# Analytical validation of a flow cytometric method for the detection and quantification of canine mast cells in peripheral blood, bone marrow, and lymph node

**DOI:** 10.3389/fvets.2025.1542460

**Published:** 2025-08-25

**Authors:** Giulia Iamone, Fulvio Riondato, Rachel Hanford, Amandine Lejeune, Amir Kol

**Affiliations:** ^1^Department of Veterinary Sciences, School of Agriculture and Veterinary Medicine, University of Turin, Grugliasco, Italy; ^2^Department of Surgical and Radiological Sciences, University of California Davis School of Veterinary Medicine, Davis, CA, United States; ^3^Department of Pathology, Microbiology and Immunology, University of California Davis School of Veterinary Medicine, Davis, CA, United States

**Keywords:** mast cell tumor, flow cytometry, canine, metastasis, lymph nodes, bone marrow, peripheral blood, analytical validation

## Abstract

Mast cell tumors (MCTs) are the most common skin neoplasms in dogs and exhibit highly variable biological behavior. Metastasis primarily affects the lymph nodes, though less frequently, MCTs can infiltrate the spleen, liver, peripheral blood, and bone marrow. Flow cytometry of fine needle aspirate samples represents a non-invasive diagnostic procedure that has shown promise for detecting and quantifying mast cells in primary tumors and lymph nodes. However, analytical validation of this method for clinical use is lacking. This study aimed to evaluate the analytical performance of a flow cytometric panel for quantifying mast cells in peripheral blood, bone marrow, and lymph node aspirates from dogs. Key parameters as the limit of blank (LOB), lower limit of detection (LLoD), lower limit of quantification (LLoQ), intra-assay precision, and accuracy, were evaluated. The method demonstrated high precision across a wide range of mast cell concentrations, with analytical coefficient of variation (CV_A_) of less than 10% for all sample types. It also showed good accuracy with minimal proportional bias observed in lymph node samples, particularly at higher mast cell concentrations. The LLoQ was 0.1% for all sample types. Flow cytometry provided reliable results highlighting its potential as a clinical tool for diagnosing and staging MCTs. These findings support the clinical applicability of flow cytometry as a minimally invasive, highly accurate method for assessing mast cell infiltration in peripheral blood, bone marrow, and lymph nodes, offering an alternative to traditional microscopic examination. This validation establishes a foundation for future studies on the prognostic implications of mast cell infiltration in MCT progression.

## 1 Introduction

Mast cell tumor (MCT) is the most common skin neoplasm in dogs (1) and its biological behavior is variable, depending on many factors such as breed (2), tumor grade (3, 4), and the presence or absence of nodal or distant metastasis. MCT primarily metastasizes to lymph nodes (5), and may later affect the spleen, liver, and other organs. Less commonly, disseminated MCT can present with mastocytemia (mast cells in peripheral blood) or mast cell leukemia (uncontrolled proliferation of mast cells in the bone marrow) (1).

Standardized histologic criteria for determining whether a lymph node is affected by metastasis was proposed by Weishaar et al. (6). This classification system divides lymph nodes into four histological node (HN) categories: HN0, non-metastatic; HN1, pre-metastatic; HN2, early metastasis, and HN3, overt metastasis. Nonetheless, histologic examination and classification according to the HN paradigm requires lymphadenectomy, an invasive surgical procedure. Cytological evaluation of lymph nodes is less invasive and less expensive but has lower sensitivity due to the lower number of cells evaluated and the potential low numbers of metastatic cells (7). Therefore, accurate pre-surgical prognostication remains a significant clinical challenge. Flow cytometry of fine needle aspirates offers a potential solution to overcome these limitations by enabling a precise quantification of these potential rare events. Indeed, Sulce and coauthors (8) described the immunophenotype of neoplastic canine mast cells and demonstrated that flow cytometry can detect and quantify mast cells in primary masses and lymph nodes as CD117+/IgE + events. In the same paper (8), the expression of CD11b was significantly associated with histopathologic grade (2-tier low-grade and 3-tier grade II), but with low sensitivity. Additionally, observed aberrancies were positivity to CD34 and CD25, but the low number of cases in the study did not allow any kind of correlation with tumor characteristics. Also, nodal infiltration of mast cells higher than 0.3% was reported as diagnostic for metastatic lymph nodes (HN2 and HN3) and a nodal infiltration higher than 4% diagnostic for HN3 nodal status (9). To date an analytical validation of this flow cytometric method is lacking while it is essential for its introduction into clinical practice.

Microscopic examination of smears from whole blood, buffy coats, and bone marrow aspirates is the current standard for detecting and quantifying mast cells in peripheral blood and bone marrow (10). However, no reports on the use of flow cytometry for this purpose are currently available.

When validating a flow cytometric method, key parameters typically assessed are: the level of blank (LOB), that is the signal detected in the absence of analyte (i.e., in blank samples); the lower level of detection (LLoD), that is the lowest level of the analyte that can be detected above the LOB; the lower level of quantification (LLoQ) which is the lowest level of analyte that can be reliably quantified. Additionally, method precision (i.e., how close the results are when the same sample is tested repeatedly under the same conditions) and accuracy (i.e., the degree of agreement between the measured result and a known value) are considered (11).

Therefore, the objective of the study is to determine the analytical performance of a flow cytometric panel for quantifying mast cells in peripheral blood, bone marrow and lymph node aspirates by assessing the LOB, LLoD, LLoQ, intra-assay precision and method accuracy as recommended by the American Association of Pharmaceutical Scientists and the International Clinical Cytometry Society (11).

## 2 Materials and methods

### 2.1 Cell culture

The NI-1^12^ canine mast cell line, established from a dog diagnosed with mast cell leukemia, was used in this study. This cell line has been used in multiple prior studies on neoplastic mast cell biology and immunotherapy (12–15). Although this line has proven useful in studying mast cell signaling and proliferation *in vitro*, data comparing its behavior to the full spectrum of *in vivo* mast cell tumors in dogs are limited. The cells were cultured in RPMI 1640 medium enriched with 10% fetal bovine serum (FBS) (R&D Systems) and 1% penicillin/streptomycin (Gibco) and incubated at 37 °C and 5% CO2. They were passed every 3 to 4 days. The cell line is positive for CD117 and IgE (after incubation with canine IgE).

### 2.2 Samples

Peripheral blood, bone marrow and lymph node samples were obtained from dogs with no history of mast cell tumor or allergic disease, admitted to the Veterinary Medicine Teaching Hospital of the University of California, Davis between March and June 2024. An IACUC protocol was approved (#23498) and an informed consent form was signed by the owner. Use of samples obtained from euthanized patients was approved by dog owner, who granted permission for a no-restriction necropsy. Animals were euthanized by qualified veterinary staff at UC Davis Veterinary Medical Teaching Hospital according to best clinical practice for clinical reasons unrelated to this study.

Peripheral blood was collected from live animals via jugular venipuncture and stored in EDTA tubes and bone marrow was collected post-euthanasia (from dogs with osteosarcoma or perianal hernia) in EDTA tubes. Lymph node samples were collected by fine needle aspiration (fenestration technique, using a 22G needle) from live animals and from warm necropsies (within 4 h from euthanasia) and placed in tubes containing 1 mL of RPMI medium enriched with 10% FBS and 1% penicillin/streptomycin. For each sample, one slide (for bone marrow and lymph nodes) or two slides (for peripheral blood) were prepared, stained with May-Grunwald Giemsa, and analyzed to ensure that mast cells constituted fewer than 1 out of 10,000 cells. Negative control samples consisted of leftover material collected for diagnostic purposes.

### 2.3 Flow cytometry

After collection, all samples were counted with an automated hematology analyzer (VETSCAN® HM5 hematology analyzer, Zoetis) to assess cellularity. This allowed to standardize the cell concentration in all tubes: 5,000 cells/μL for peripherl blood and 1,000 cells/μL for lymph nodes and bone marrow. NI-1 canine mast cells have IgE receptors, but in the absence of canine IgE, NI-1 cells are negative when stained with an anti-canine IgE antibody (Supplementary Figure 1). To ensure IgE positivity, the NI-1 cells were pre-incubated with dog serum at 37 °C for 2 h (0.5 mL of serum in 1 mL of NI-1 cell suspension) prior to flow cytometric analysis.

**Figure 1:**
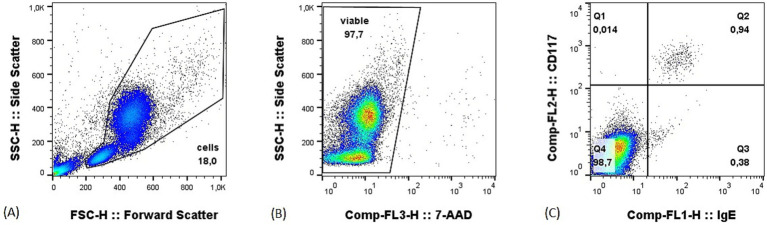
Example of gating strategy on a peripheral blood sample with 1% of mast cells. **(A)** Morphological gate in the forward (FSC) versus side (SSC) scatter; **(B)** viability gate set to exclude dead cells; **(C)** IgE versus CD117 plot, mast cells are the double positive events in the upper right quadrant.

All samples were analyzed within 24 h from collection. After mast cells were spiked into the matrices, 1uL of polyclonal anti-canine IgE-FITC (Bio-Rad) and 0.5uL of anti-mouse CD117-PE (clone ACK45, BD pharmingen) antibodies were added. A total of cells 2 million per tube were used for peripheral blood samples, and 500,000 cells per tube for bone marrow and lymph nodes. Tubes were then incubated at 4 °C in the dark for 15 min. Following an RBC lysis step, a viability dye (5 uL of 7AAD, Invitrogen) was added, and the samples resuspended in 200 μL of PBS. All samples were then acquired on a FACScalibur cytometer (BD Biosciences). A minimum of 500,000 events for peripheral blood and of 100,000 events for lymph nodes and bone marrow samples were recorded in the “cells” gate (Figure 1). The gating strategy consisted of a morphological gate in the FSC versus SSC scatter to exclude debris and events smaller than lymphocytes (i.e., “cells”), followed by a viability gate to exclude dead cells. Finally, an IgE versus CD117 plot was used to identify mast cells as the percentage of double positive events (Figure 1). Flow cytometric data were analyzed using a commercially available software (Flowjo^™^).

### 2.4 Experimental design

The appropriate quantity of mast cells from the NI-1 cell line (16) was added to peripheral blood, bone marrow and lymph node samples in order to obtain the following mast cell concentrations: 10, 7, 5, 3, 2, 1, 0.5, 0.3 and 0.1% in peripheral blood and bone marrow, and 50, 25, 10, 5, 3, 1, 0.5, 0.3 and 0.1% in lymph nodes. Samples without added mast cells were used as negative controls. The number of samples, repeats run, and dilutions analyzed are reported in Table 1.

**Table 1:** Samples, repeats and dilutions used for each assessed parameter and how they were calculated.

Parameter	Samples	Repeats	Dilutions	Calculation (11)	Acceptable CV_A_ (11)/expected values
LOB	10	1	-	Mean + 1.65 SD	-
LLoD	10	1	-	LOB + 1.65 SD	-
LLoQ	3	3	5^§^	Lowest concentration ≥ LLoD Values proportional to the dilution scheme applied	30–35%
Overall precision	3	3	9^†^	Overall CV_A_ = $\sqrt{mean\;}ofCV_{A}^{2}$	10–25%
Accuracy	3	1	9^†^	Regression analysis (Passing-Bablock)	Intercept = 0 Slope = 1

Last column reports acceptable values, when appropriate. LOB, level of blank, LLoD, lower level of detection, LLoQ, lower level of quantification, SD, standard deviation, CV_A_, analytical coefficient of variation. § Dilutions were 2, 1, 0.5, 0.3 and 0.1% for peripheral blood and bone marrow; 3, 1, 0.5, 0.3 and 0.1% for lymph nodes. †Dilutions were 10, 7, 5, 3, 2, 1, 0.5, 0.3 and 0.1% for peripheral blood and bone marrow; 50, 25, 10, 5, 3, 1, 0.5, 0.3 and 0.1% for lymph nodes.

### 2.5 Statistical analysis

LOB, LLoD, LLoQ, intra-assay precision and accuracy were calculated as described in Selliah et al. (11). LOB was calculated as the mean of negative control outputs plus 1.65 SD (standard deviation) and LLoD was defined as the LOB plus 1.65 SD. The value selected as LLoQ was the highest dilution (i.e., lowest mast cell concentration) above the LLoD with an analytical coefficient of variation (CV_A_) < 30% based on 3 repeats. Overall precision was calculated as the square root of the mean of all the squared CV_A_s of the different dilutions, with acceptable value for overall CV_A_ set at < 10%. Passing-Bablock regression was performed to assess accuracy. The fit was considered good when the 95% confidence interval for the intercept included zero, and the 95% confidence interval for the slope included one. Statistical analysis was run on Analyse-it for Excel (Microsoft Corporation, Redmond, WA, United States).

## 3 Results

Thirteen peripheral blood samples, 5 bone marrow samples and 15 lymph node samples were collected from 22 different dogs. Peripheral blood samples were obtained from routine pre-ovariohysterectomy screening (*n* = 2), routine check-ups for vaccination or preventative administration (*n* = 2), blood donors (*n* = 2), dogs with ocular lesions (*n* = 2), kidney disease (*n* = 2), esophageal stricture (*n* = 1), high-grade B-cell lymphoma (*n* = 1) and prostatic carcinoma (*n* = 1). Bone marrow samples came from warm necropsies of dogs with osteosarcoma (*n* = 4) or perianal hernia (*n* = 1). Lymph node samples came from dogs with multicentric lymphoma (*n* = 3) or osteosarcoma (*n* = 3).

**Figure 2:**
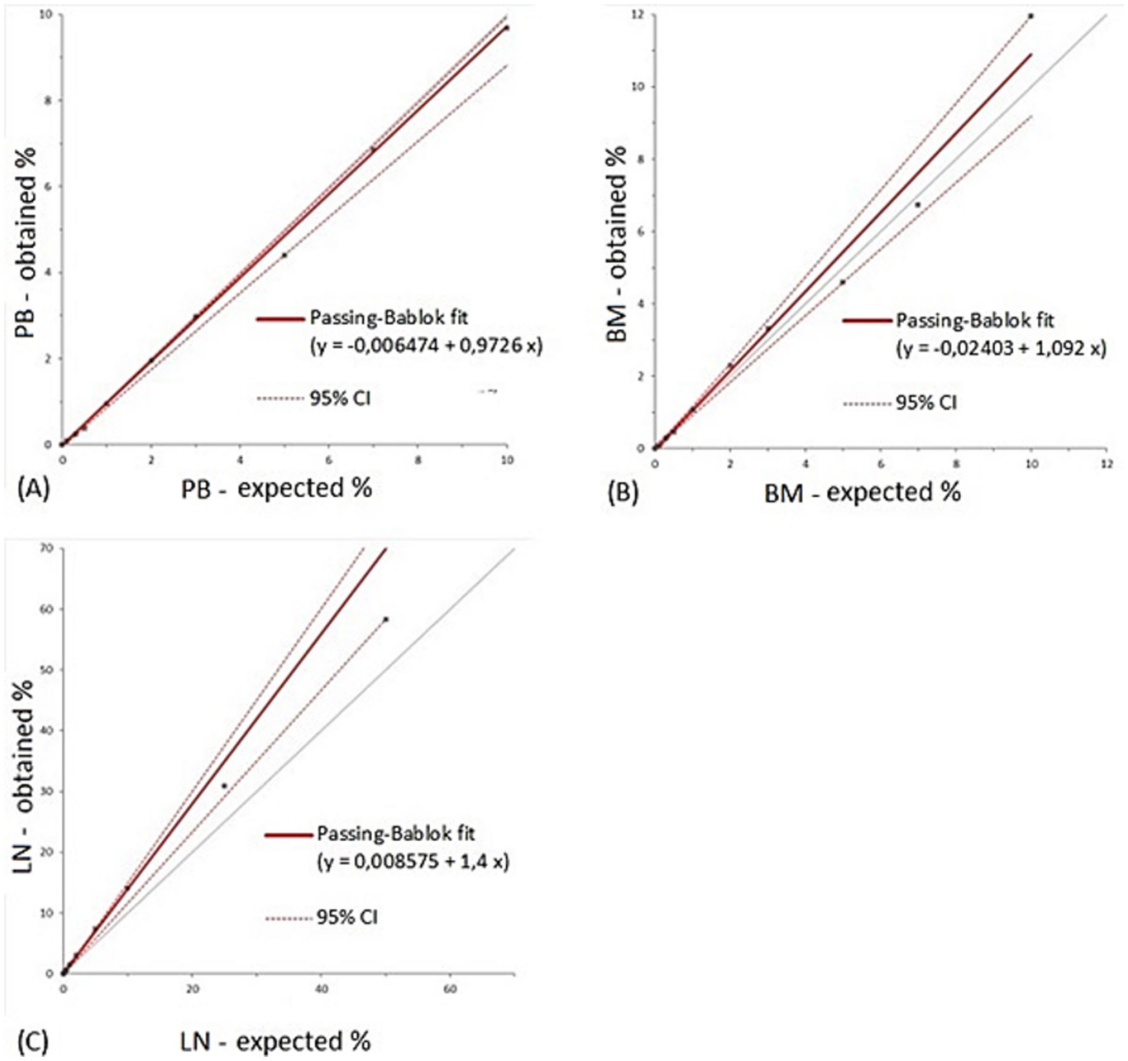
Results from Passing-Bablock regression analysis for peripheral blood **(A)**, bone marrow **(B)** and lymph node **(C)**. The grey line represents the identity line. PB, peripheral blood; BM, bone marrow; LN, lymph node; CI, confidence interval.

LOB was 0.007% for peripheral blood, 0.013% for bone marrow and 0.025% for lymph node negative controls (Table 2). LLoD was calculated as 0.010% for peripheral blood, 0.021% for bone marrow and 0.042% for lymph node. The value selected as LLoQ was 0.1% for all matrices. Overall precision was considered clinically acceptable in all matrices with CV_A_s below 10%: 5.7% for peripheral blood, 9% for bone marrow and 8.4% for lymph node. CV_A_ values for each dilution tested for the three matrices are reported in Table 3. Notably, for the previously reported cut-off of 0.3% of nodal infiltration (9), the CV_A_ is of 11.93%; whereas for infiltrations greater than 0.5%, the CV_A_ remained below 10%. Passing-Bablock analysis (Figure 2) revealed no constant or proportional bias between expected and measured percentages in peripheral blood (intercept −0.007, 95% CI −0.064 to 0.013; slope 0.973, 95% CI 0.881 to 1) and bone marrow (intercept −0.024, 95% CI −0.137 to 0.091; slope 1.092, 95% CI 0.917 to 1.205). However, a slight proportional bias was observed in lymph node samples (intercept −0.009, 95% CI −0.051 to 0.687; slope 1.4, 95% CI 1.167 to 1.504) leading to an overestimation of mast cell nodal infiltration at higher percentages. All results are summarized in Table 2.

**Table 2:** Values of level of blank (LOB), lower level of detection (LLoD) and quantification (LLoQ), overall precision and accuracy of the method for each matrix.

Parameter	PB	BM	LN
LOB	0.007%	0.013%	0.025%
LLoD	0.010%	0.020%	0.042%
LLoQ	0.1% (CV_A_ = 12.3%)	0.1% (CV_A_ = 13.4%)	0.1% (CV_A_ = 9.5%)
Overall precision	CV_A_ = 5.7%	CV_A_ = 9%	CV_A_ = 8.4%
Accuracy	Intercept = −0.007 (CI95%: −0.064 to 0.013) Slope = 0.973 (CI 95%: 0.881 to 1)	Intercept = −0.024 (CI95%: −0.137 to 0.091) Slope = 1.092 (CI 95%: 0.917 to 1.205)	Intercept = 0.009 (CI95%: −0.051 to 0.687) Slope = 1.4 (CI 95%: 1.167 to 1.504)

PB, peripheral blood; BM, bone marrow; LN, lymph node; CV_A_, coefficient of variation; CI, confidence interval.

**Table 3:** Precision for each dilution and each matrix.

Dilution	PB (CV_A_ %)	BM (CV_A_ %)	LN (CV_A_ %)
50%	-	-	0.57
25%	-	-	8.94
10%	1.79	2.54	5.45
7%	1.65	7.42	-
5%	4.14	4.62	4.07
3%	4.07	4.27	-
2%	3.71	3.61	9.37
1%	4.42	9.83	9.37
0.5%	5.58	11.31	9.99
0.3%	6.14	13.77	11.93
0.1%	12.30	13.39	9.50

Each CV_A_ value is calculated out of 3 repeats. PB, peripheral blood; BM, bone marrow; LN, lymph node; CV_A_, coefficient of variation.

When addressing the uncertainty that occurs when measuring any parameter, we underline that in lymph nodes [the only matrix for which diagnostic cut-offs are reported (9)], the calculated value of extended measurement uncertainty (EMU) (17), based on the obtained CV_A_ (8.4%), is of 0.17. Thus, when applying the present flow cytometric protocol, obtained values of nodal infiltration should be reported as the measured result (x) ± the uncertainty level according to the following equation: x ± (x · EMU). For example, the 0.3% value should be reported as 0.3 ± 0.05%.

## 4 Discussion

This study describes the performances of flow cytometry as an analytic tool for the detection and quantification of canine mast cells in peripheral blood, bone marrow and lymph nodes in a clinical setting. Based on our results, flow cytometry can reliably detect and quantify mast cells in peripheral blood, bone marrow and lymph nodes with excellent precision and accuracy across a wide range of clinically relevant mast cell concentrations. We observed a proportional bias in mast cell quantification in lymph nodes, which indicates an increasing over-detection of mast cells as their concentration rises within lymph node samples. However, this bias is of minimal clinical concern in the clinically relevant range of mast cell concentration which is 0.3% to discriminate non metastatic lymph nodes from metastatic ones and 4% to identify HN3 lymph nodes (9).

When assessing the analytical precision of the method, we observed that overall precision was consistently below 10% for all matrices, which is considered acceptable (11). The precision of the method decreased at the 0.3% cut-off for lymph node infiltration but remained acceptable (< 25%) since rare events are targeted (11). For higher percentages of mast cell infiltration, individual CV_A_s remained below 10%. LOB and LLoD values were low, indicating very few false positive events and high sensitivity of the method, respectively.

Despite we acquired a lower number of events for bone marrow and lymph nodes compared to peripheral blood, the flow cytometric method still demonstrated good precision and low LLoQ across all matrices. This suggests that flow cytometry can provide reliable results even when working with sub-optimal sample quality. However, it is strongly recommended to acquire a high number of events when looking for low percentages of target cells to maintain reliable measurements. In this study, we analyzed 100,000 cells and the precision for the lowest measured mast cell percentage (0.1%) in lymph node samples was 9.5%. A lower precision would be expected if fewer cells were acquired and analyzed.

Sample size may affect the reliability of LOB and LLoD calculations for bone marrow, as we had fewer negative controls (*n* = 5) compared to other matrices. This was due to the challenges in obtaining fresh bone marrow samples, which are more invasive to collect than peripheral blood or lymph node aspirates. Despite this limitation, the sample size was sufficient for evaluating the other parameters (LLoQ, precision and accuracy). Also, LOB and LLoD for bone marrow samples remained well below the clinically relevant threshold (9).

While CD117 is the antibody that most consistently identifies mast cells in MCTs (8), we added IgE as a second marker to increase specificity for the mast cell population we aimed to quantify. Canine mature basophils show positivity to IgE (18), but are immunohistochemically negative to c-kit (19). Basophil precursors have been described as IgE + and CD117 + in humans (20), but their immunophenotype in dogs is unknown. Therefore, they could potentially show, in bone marrow, an immunophenotype that overlaps with mast cells. Nevertheless, they usually represent a very small proportion of cells in bone marrow and, therefore, this population could be of minor concern when quantifying mast cells in this matrix. The use of IgE is also valuable for identifying aberrant phenotypes (CD117 + IgE-) that may occur in neoplastic mast cells (8), helping to detect these cells more easily across all sample types.

In conclusion, we observed that flow cytometry is a viable and reliable clinical method for detecting and quantifying mast cells in peripheral blood, bone marrow and lymph nodes, matrices that are likely to be infiltrated by this neoplasm. Therefore, flow cytometry could be adopted as a standard procedure in the diagnosis and staging of canine MCT. This is particularly relevant for peripheral blood and bone marrow, where the only current alternatives (microscopic examination of blood smears or buffy coat slides) are limited by the small number of cells that can be analyzed, particularly when compared to a flow cytometric analysis, and are highly dependent on operator skill and staining techniques (21). Moreover, while we have observed a proportional bias in lymph node samples, flow cytometry remains a superior tool compared to manual quantification, particularly within the clinically relevant mast cell range of 0.3 to 4%. Our findings validate the tested flow cytometric panel, further supporting the previously reported diagnostic cut-offs and providing a foundation for possible future studies on the prognostic significance of mast cell infiltration in different matrices.

## Data Availability

The original contributions presented in the study are included in the article/Supplementary material, further inquiries can be directed to the corresponding author.

## Supplementary material

SUPPLEMENTARY FIGURE 1 **(A)** NI-1 cells not labelled (left) and labelled with IgE-FITC (right) before incubation with dog serum. Less than 6% of the events were IgE positive. **(B)** NI-1 cells not labelled (left) and labelled with IgE-FITC (right) after a 2 hour incubation step with dog serum. More than 97% of cells were IgE positive.
