# Supplementary figures and images for: Analytical validation of a flow cytometric method for the detection and quantification of canine mast cells in peripheral blood, bone marrow, and lymph node
Source: Front Vet Sci. 2025 Aug 25;12:1542460. doi: 10.3389/fvets.2025.1542460 (PMC12414779; doi:10.3389/fvets.2025.1542460)

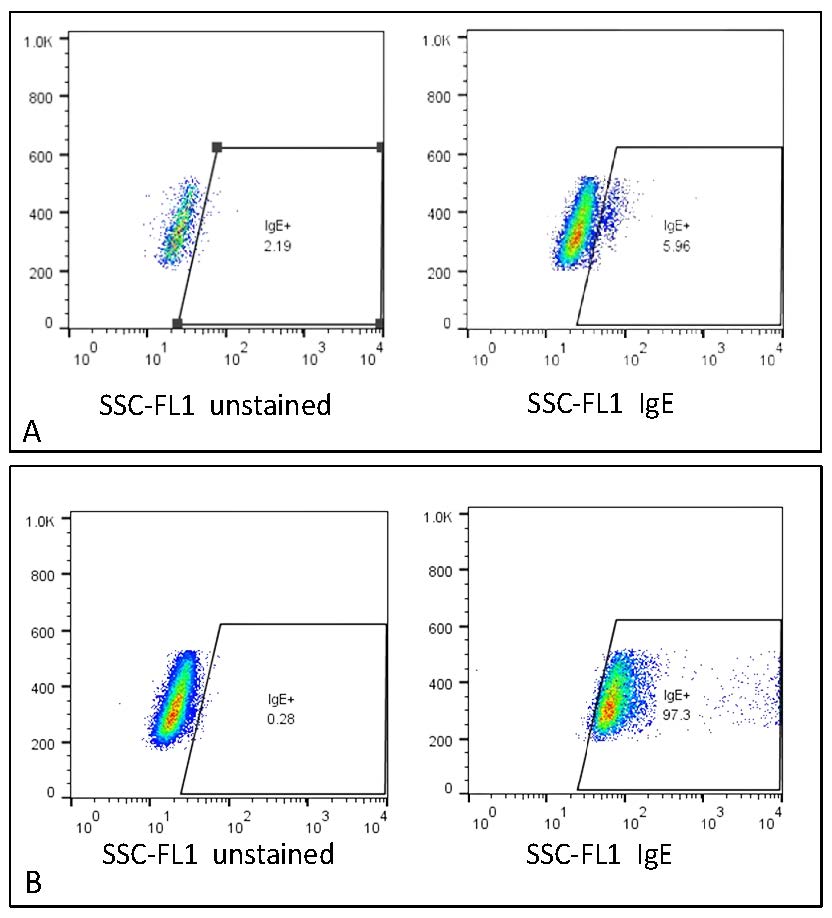

Supplement: SUPPLEMENTARY FIGURE 1 — (A) NI-1 cells not labelled (left) and labelled with IgE-FITC (right) before incubation with dog serum. Less than 6% of the events were IgE positive. (B) NI-1 cells not labelled (left) and labelled with IgE-FITC (right) after a 2 hour incubation step with dog serum. More than 97% of cells were IgE positive. [file Image_1.jpeg]
